# Vascular Endothelial Growth Factor as a Putative Biomarker of Depression in Asthmatics with Reversible Airway Narrowing

**DOI:** 10.3390/jcm10225301

**Published:** 2021-11-15

**Authors:** Krzysztof Gomułka, Jerzy Liebhart, Wojciech Mędrala

**Affiliations:** Department of Internal Medicine, Pneumology and Allergology, Wroclaw Medical University, 50-369 Wroclaw, Poland; jerzy.liebhart@umed.wroc.pl (J.L.); wojciech.medrala@umed.wroc.pl (W.M.)

**Keywords:** asthma, biomarker, depression, ELISA-based detection, vascular endothelial growth factor

## Abstract

The vascular endothelial growth factor (VEGF) plays a pivotal role in process of angiogenesis in adults. If angiogenesis is not properly controlled, its deregulation may implicate it in various psychosomatic diseases states. The aim of our study was to reveal possible correlation between severity of depression in asthmatics with different degrees of airway narrowing and serum vascular endothelial growth factor levels. The study population included a total of 122 adult subjects: 82 patients with asthma (among them 42 patients with irreversible bronchoconstriction and 40 patients with reversible bronchoconstriction) and 40 healthy participants as a control group. The standardized Beck Depression Inventory (BDI) was used to estimate the depression symptoms. Enzyme-linked immunosorbent assay (ELISA) was used to assess the VEGF serum concentration in all participants. There was a significant difference in depression symptoms in asthmatics with reversible (*p* = 0.0432) and irreversible airway obstruction (*p* = 0.00005) in comparison to control group and between these two subgroups of asthmatics (*p* = 0.0233). Obtained results revealed significant correlation between level of depression and mean VEGF serum concentration in asthmatics with reversible airway obstruction (*p* = 0.0202). There was no difference between enhanced depression symptoms and VEGF serum concentration in patients with irreversible airway obstruction nor in the total group of asthmatics (in both *p* > 0.05). The relationship between asthma severity and depression symptoms seems to be certain. VEGF might be considered as a putative biomarker of depression in asthmatics, mainly those with reversible airway narrowing.

## 1. Introduction

The vascular endothelial growth factor (VEGF) has been identified to play a main role in regulation of angiogenesis processes. This 45 kDa basic heparin-binding homodimeric glycoprotein is expressed in epithelial cells, neutrophils, platelets, and macrophages. There are several members of this not homogeneous “VEGF family”: VEGF-A (commonly referred to as VEGF), VEGF-B, VEGF-C, VEGF-D, VEGF-E, VEGF-F, and placental growth factor (PlGF). VEGF-A is the protypical member with four splice variants that are identified as follows: VEGF 115, 120, 164, and 188. Their biological activity is dependent on binding with tyrosine kinase receptors (VEGFR-1, VEGFR-2, or VEGFR-3) that are located on vascular and lymphoid endothelial cells [1,2]. VEGF-A can bind to VEGFR-1 (flt-1) and VEGFR-2 (KDR/flt-1) receptors. The VEGFR-2 receptor mediates nearly all known types of cellular response to the action of VEGF. The function of the VEGFR-1 receptor is less known so far—it probably exerts a regulatory effect as the so-called “Trap receptor” and modulates the binding of vascular endothelial growth factor to VEGFR-2, which may be particularly important in the formation of a network of blood vessels during embryonic development. VEGFR-3, another VEGF receptor, does not bind VEGF-A and aims to mediate lymphangiogenesis; its ligands, in turn, are VEGF-C and VEGF-D. As the proangiogenic element associated with hypoxia-inducible factor (HIF-1α) path, VEGF stimulates proliferation and maturation of endothelial cells and through this regulates the angiogenic balance in both embryonic and adult stages. If the angiogenesis is not properly controlled, its deregulation may implicate it in various diseases states such as cancers and metastasis, ischemic heart disease, ulcers, sepsis, age-related macular degeneration, rheumatoid diseases, and chronic obstructive pulmonary disease. For this reason, VEGF might be considered as a biomarker of vascular remodeling. Moreover, the ability to regulate VEGF synthesis and activity is of interest in being a target for new therapies [3,4,5]. VEGF-related therapeutic strategies may be the use of humanized anti-VEGF-A monoclonal antibodies (bevacizumab), a soluble form of a protein fusion of the extracellular VEGFR domain and IgG Fc region (called VEGF-Trap-ranibizumab) or signal inhibition transmitted by VEGFR with VEGF receptor tyrosine kinase inhibitors (sunitinib, sorafenib, axitinib).

Considering respiratory tract diseases, VEGF as the strongest regulator of blood vessel growth, initiates the changes in bronchial microvasculature that contribute to the airway remodeling in chronic bronchial inflammation in asthma. Higher expression of VEGF may also promote proliferation of airway smooth muscle cells, up-regulate disintegrin and metalloproteinase (ADAM-33) mRNA, enhance allergic sensitization with T-helper-2 type inflammatory responses, and enhance chemotaxis for monocytes and eosinophils [6,7]. VEGF has been previously reported as biomarker of asthma exacerbation [8]. Moreover, VEGF serum concentration has been reported to be higher in patients with asthma than in healthy persons, and has been noticed to be the highest in asthmatics with irreversible airway narrowing when the presence of depression is not taken into account [9].

Additionally, VEGF can influence some neural processes in the adult brain. This molecule induces neural progenitor cell division, stimulates adult neurogenesis in vitro and in vivo, and promotes neurite outgrowth and maturation during development. Moreover, the role of VEGF in motor neuron degeneration, tumorigenesis, or ischemia in the central nervous system connected with stroke suggests that it might be a therapeutic target for the treatment of various neurological disorders [10,11]. It is possible that VEGF is acting on specific areas of the adult brain, such as neurogenic subventricular zone and subgranular zone in the hippocampus, where neurogenesis is closely linked with ongoing angiogenesis. Dysfunction on these fields in the hippocampus is found, at least in part, in patients with depression. The neurotrophic hypothesis of depressive disorder postulates that this illness results from aberrant neurogenesis in brain regions that regulates emotion and memory. It suggests that VEGF as a trophic factor that connects angiogenesis and neurogenesis may also be involved in pathophysiology of depression [12,13]. Depression is a disabling mental disease that affects many people of all ages worldwide—the World Health Organization estimates that by the end of current decade, depression will become the leading cause of lost disability-adjusted life years and is ranked as the highest global cause of “years lived with disability” [14,15].

Reviewing the updated literature, it seems that data on possible biochemical markers of depression in asthmatics are lacking. Therefore, the main objective of this study was to contribute the correlation between severity of depression in asthmatics with different degree of airway narrowing and serum vascular endothelial growth factor level.

## 2. Materials and Methods

### 2.1. Study Groups

This study included a total of 122 subjects (42 males; aged from 20 to 70 years) who gave written and informed consent for participation. Among the study group, 82 patients (28 males; aged from 23 to 69 years) had the diagnosis of asthma established earlier according to The Global Initiative for Asthma (GINA) recommendation [16]. Based on standard bronchodilation test with salbutamol, asthmatics were divided into two cohorts: 40 patients with reversible airway obstruction (11 males; aged from 23 to 69 years) and 42 patients with irreversible airway obstruction (17 males; aged from 24 to 69 years). The predicted post-bronchodilator values of FEV1 < 80% and FEV1/FVC < 70% were taken as a criterion of irreversible airway obstruction. Exclusion criteria were age under 18 or over 70 years, asthma exacerbation, asthma, chronic obstructive pulmonary disease overlap (ACO), substance use disorder, and the coexistence of severe chronic diseases (neoplasm, etc.). In asthmatics with reversible airway narrowing, 27 participants used inhaled corticosteroids (budesonide or fluticasone propionate), and 28 patients were treated with long-acting beta-2 agonists (salmeterol or formoterol fumarate). Among asthmatics with irreversible airway obstruction, 37 participants used inhaled corticosteroids (budesonide or fluticasone propionate), and 38 patients were treated with long-acting beta-2 agonists (salmeterol or formoterol fumarate). None of the patients participating in our study declared the use of antidepressants or systemic corticosteroids.

The control group consisted of 40 healthy volunteers (14 males; from 20 to 70 years) without allergies or chronic pulmonary dysfunction. The characteristic of the examined groups is summarized in Table 1.

### 2.2. Severity of Depression

The Beck Depression Inventory (BDI) contained 21 questions and was used to assess the severity of depression [17,18]. Each question had four statements reflecting varying intensities of symptoms, which were scored from 0 to 3 points, respectively. According to the standard cut-off, depression might be estimated as no signs of depression (0–11 points), mild to moderate level of depression (12–27 points), and very high level of depression (28 points and above).

### 2.3. Blood VEGF Levels in Examined Participants

To estimate the mean VEGF serum concentration in (pg/mL), venous blood samples were taken from all participants. Individual blood samples were assayed twice, and the average value was taken for further analysis. The evaluations were performed with an enzyme-linked immunosorbent assay (ELISA) by the Quantikine Human VEGF Immunoassay kit, where the normal level of VEGF serum concentration was from 62 to 707 pg/mL (following the manufacturer’s instructions). The minimum detectable VEGF concentration was no less than 5.0 pg/mL.

### 2.4. Statistical Analysis

Statistical analysis was performed using the Statistica 12 and Epi Info 7.2.3.1. software. The Bartlett test was used to examine the homogeneity of variance. Verification of the hypothesis of equality of mean parameters in independent groups was carried out by ANOVA, or for groups with heterogeneous variance by the nonparametric Mann–Whitney U test. The χ^2^ test was used to compare nominal and ordinal data. The Spearman correlation coefficient was performed for analysis of selected parameter pairs. Statistical significance was set at *p* < 0.05.

## 3. Results

**Figure 1:**
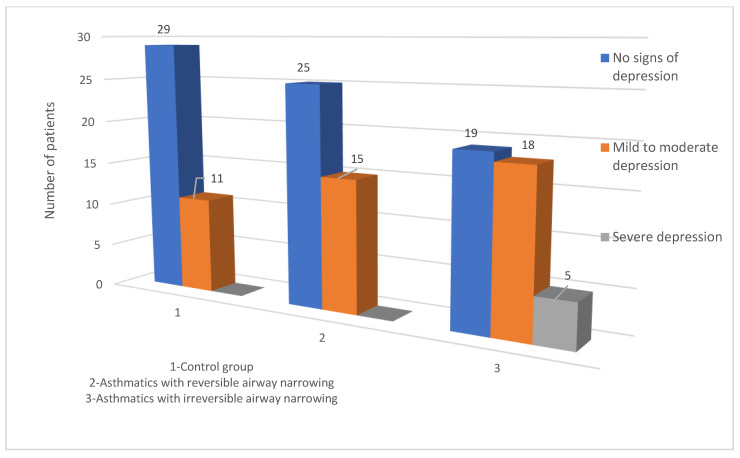
Number of participants with depression among the examined groups.

The level of depression was higher among patients with asthma than in control group. A mild to moderate level of depression was observed in 15 (37.5%) asthmatics with reversible airway narrowing, in 18 (42.86%) asthmatics with irreversible airway obstruction, and 11 (27.5%) participants from the control group. In asthmatics with irreversible airway obstruction, five patients (11.9%) had a very high level of depressive disorder, which was not detected in patients with reversible broncho-obturation nor in the controls (Figure 1). There was a statistically significant difference in the mean level of depression in asthmatics with reversible and irreversible airway obstruction in comparison to the control group (*p* = 0.0432 and *p* = 0.00005, respectively) and between the two subgroups of asthmatics (reversible vs. irreversible airway narrowing; *p* = 0.0233).

Moreover, the VEGF serum concentration was evaluated in study groups with a different severity of depression symptoms. There was a statistically significant difference in VEGF expression in asthmatics with reversible and irreversible airway obstruction in comparison to the control group (*p* = 0.045 and *p* = 0.046, respectively) among participants with mild to moderate enhanced depression (Table 2).

Additionally, this studied sample showed a statistically significant correlation between the level of depression and VEGF serum concentration in asthmatics with reversible airway narrowing (*p* = 0.0202). There was no statistically significant correlation (*p* > 0.05) between the severity of depressive disorders and mean VEGF serum concentration in patients with irreversible broncho-obturation in the total group of asthmatics or in the control group (Table 1).

## 4. Discussion

There is strong evidence that various cytokines contribute to the symptoms of depression by affecting neurotransmission, neuroplasticity, and neuroendocrine processes. Additionally, depression is considered as an indicator of poor outcomes in several chronic diseases including asthma, which was linked to psychosomatic pathology and even included to the group of the “holy seven” psychosomatic illnesses by Alexander [19]. In the clinical course of asthma, chronic airway inflammation and limitations in airflow are the main aspects of this heterogeneous disease. On the other hand, depression is cited as factor affecting patients’ subjective evaluation of asthmatic symptoms; it reduces the health-related quality of life, worsens asthma management, correlates with asthma severity, and affects the asthma control status and treatment adherence [20,21]. Some findings suggest that adult patients with depressive symptoms may have a nearly twice higher risk of asthma onset than those without depression [14,22]. A longitudinal observational study by Brunner et al. [23] points to depression as a marker of risk for incident adult-onset asthma in middle-aged adults. The survey by Brown et al. [24] conducted in a 12-week, randomized, double-blind, placebo-controlled trial of patients with severe asthma and depression showed that escitalopram (SSRI—selective serotonin reuptake inhibitor) added to the therapy may be associated with reduction in the Asthma Control Questionnaire (ACQ), oral corticosteroid use, and reduction in the Inventory of Depressive Symptomatology–Self-Report (IDS-SR). Thus, better controlling of depression may lead to newer preventative strategies for asthma in adults.

Our study revealed the tendency of a higher level of depression symptoms in asthmatics in comparison to the healthy control group. Additionally, in regard to the above tendency, it was shown that the highest level of depression occurred in asthmatics with irreversible airway obstruction. Our observation on this field might relate to asthma severity, limitation of physical activity, and interpersonal contacts in this group of asthmatics, as well as possible frequent exacerbations and hospitalization. Systemic glucocorticosteroid use in treatment according to GINA recommendations [16] should also be taken into account, because steroids themselves can induce depressive disorders [25,26]. The coexistence of asthma and depression is a complex issue, which can disrupt patient–physician contacts, worsen the control of asthma, and decrease the quality of life. In some cases of patients, especially those with severe asthma and irreversible airway narrowing, the cooperation between allergists and psychiatrists, as far as antidepressant therapy is concerned, seems to be necessary. One of the most widely used psychometric tests for measuring the severity of depression is the Beck Depression Inventory (BDI). It is a 21-question multiple-choice self-report inventory developed by Aaron Beck and published in 1961 and revised in 1978 and 1996. The BDI is a helpful assessment tool of depressive disorders and their intensity for individuals aged 13 and over. This questionnaire includes features representing various aspects or symptoms of depression such as guilt feelings, questions concerning physical conditions (tiredness, loss of body mass, or loss of interest in sexual activity), hopelessness and irritation, and severity of the cognitive impairment [17,18]. The BDI might be a valid and reliable instrument to measure depression in patients with chronic diseases including asthma [27,28,29]. Diagnoses of psychiatric states primarily based on relatively subjective assessment of symptoms need to be supported by a noninvasive, blood-based biomarker panel that accurately reflects pathological processes in these disorders. The definition says that biomarkers are objectively measured and evaluated as indicators of normal biological processes, pathogenic processes, or pharmacologic responses to a therapeutic intervention. Biomarkers should also provide high levels of sensitivity and specificity in the detection and classification of disease to be clinically useful [30].

According to this, in our paper, we decided to consider peripheral VEGF as an interesting candidate to be a biochemical marker of depression in asthmatics. Our results are mixed: only in the group with reversible airway narrowing did we find that the VEGF level was significantly correlated with enhanced depression symptoms, which could not be demonstrated either in patients with irreversible airway obstruction or in the total group of asthmatics. The current body of literature features data on a wide variety of possible biomarkers linked to depressive disorders in both adults (interferon-γ, monocyte chemotactic protein-1, macrophage inflammatory protein-1α, matrix metalloproteinase-2, tumor necrosis factor-α, hepatocyte growth factor, insulin polypeptides, pregnancy-associated plasma protein A) and adolescence (interleukin-6) [31,32,33]. The available results of clinical studies focused on VEGF as a candidate plasma biomarker of depression in adult patients are ambiguous. Most of them found mRNA expression and peripheral VEGF levels elevated in relation to depressive symptoms in examined individuals, and this tendency can be reversed with antidepressant treatment [30,32,34,35,36]. It could be concluded that VEGF may still hold promise as a biomarker of mood disorders, especially considering asthmatics with a different severity of airways obstruction.

The strong point of our survey is that it was the first study in asthmatics determining the relationship between VEGF concentration and depression, and was conducted on quite large and well-matched group of patients with different degrades of airways narrowing. However, several limitations should be noted. Firstly, the symptoms of depression were estimated based on a questionnaire that was constructed by self-reported rating scales, and those scores can be exaggerated or decreased by the person completing them. It might be important that a clinical team, such as a psychologist, psychiatrist, or support care team’s consultation for the patient’s observation, should be incorporated in establishing the determination of enhanced depression. Depressive symptoms might be assessed by other useful item, i.e., the Hamilton Depression Rating Scale (HDRS). Furthermore, another bias may be that only one possible biomarker (VEGF) was chosen and tested in asthmatics. In planning future investigations about the neurotrophic hypothesis of depressive disorder among a bigger cohort, more biomarkers (for example, blood eosinophilia and brain-derived neurotropic factor—BDNF) or clinical tests (for example, flow cytometry) should also be included to identify certain immunology markers.

## 5. Conclusions

In this manuscript we explore the link between physical health and mental health. Here, the link between depression and worsening asthma conditions in patients was chosen as an interesting area to study. It is very well known that physical well-being might have a detrimental impact on patients’ mental health. Additionally, many research studies have been published to show that asthma symptoms may be directly related to mental health conditions. Relationship between asthma severity and depression symptoms seems to be certain and well proven. While there is no single factor that influences brain function and behavior, and laboratory diagnostic tools remain elusive, VEGF might be considered as a putative biomarker of depression in asthmatics with reversible airway narrowing. Our findings may be useful in investigations of pathophysiological mechanisms of depression and as targets of novel treatment approaches in an era of personalized medicine.

## Figures and Tables

**Table 1:** The study population data.

Variables	Asthmatics		Controls
	Reversible Airway Narrowing	Irreversible Airway Narrowing	
Number of participants (N)	40	42	40
Male gender (%N)	11 (27.5%)	17 (40.5%)	14 (35.0%)
Age (years) $\bar{x}\;\pm \;\mathrm{SD}$	50.0 ± 13.7	53.7 ± 10.4	48.0 ± 13.7
Age (years) Me (min; max)	52.0 (23.0; 69.0)	55.0 (24.0; 69.0)	52.5 (20.0; 70.0)
Asthma duration (mean years)	21.94	10.04	-
inhGKS (%N)	37 (88.1%)	27 (67.5%)	-
LABA (%N)	38 (90.48%)	28 (70%)	-
BDI (points) $\bar{x}\;\pm \;\mathrm{SD}$	10.2 ± 7.3 *^#^	15.2 ± 10.6 *	6.88 ± 7.16 *
BDI (points) Me (min; max)	8.0 (0.0; 25.0)	13.5 (0.0; 44.0)	4.5 (0.0; 23.0)
VEGF (pg/mL) $\bar{x}\;\pm \;\mathrm{SD}$	379.6 ± 249.9 ^#^	430.8 ± 306.6	274.9 ± 171.5
VEGF (pg/mL) Me (min; max)	288.6 (71.7; 1134.0)	340.7 (85.9; 1470.0)	246.6 (57.3; 714.7)

N—number of participants, $\bar{x}$—mean, SD—standard deviation, Me (min; max)—median (minimum; maximum), inhGKS—inhaled corticosteroids, LABA—long-acting beta-2 agonists, BDI—Beck Depression Inventory, VEGF—vascular endothelial growth factor, *—significance level of *p* < 0.05 (comparison between groups), **^#^**—significance level of *p* < 0.05 (Spearman correlation coefficient).

**Table 2:** VEGF serum concentrations ($\bar{x}\;\pm \;\mathrm{SD}$ in (pg/mL)) among the examined groups with different levels of depression.

Severity of Depression (Based on BDI)	Asthmatics		Controls
	Reversible Airway Narrowing	Irreversible Airway Narrowing	
No signs of depression	320.58 ± 191.76	486.49 ± 401.57	298.56 ± 185.76
Mild to moderate depression	478.04 ± 307.11 ^#^	403.44 ± 206.63 *	212.55 ± 110.53 *^#^
Severe depression	-	318.08 ± 149.99	-

VEGF—vascular endothelial growth factor, $\bar{x}$—mean, SD—standard deviation, BDI—Beck Depression Inventory, *—significance level of *p* < 0.05 (comparison between groups), **^#^**—significance level of *p* < 0.05 (comparison between groups).

## Data Availability

The data that support the findings of this study are available from the corresponding author upon request.
